# Report of the Diseases of Edinburgh for July, 1809

**Published:** 1809-09

**Authors:** John Roberton


### *Report of the Diseases of Edinburgh for July,* 1809. By John Roberton, M. D.

For several days, about the beginning of the month, the weather was moist, and there were occasional falls of rain. This was succeeded by some days of pleasant and agreeable weather; but these again being succeeded by easterly winds, fogs of greater or less density, especially after sunset, overspread the neighbourhood of the city. This sort of weather, intermixed with occasional good days, continued till about the middle of the month, when we had, for a day or two, considerable gales of wind. Several days of pleasant weather then intervened, but easterly winds and fogs succeeding them, rendered the weather damp and disagreeable. We had again some gales of wind, succeeded by thunder and showers of rain, after which, till the termination of the month, the weather continued very fine.

During the first third of the month, the barometer was rather low, but after that till about the middle of it, it began to rise. From this time till the end of the month, the barometer varied considerably, but never was very high.

The thermometer also varied considerably. In the good days, it rose high, sometimes beyond 70°, at other times, especially at night, and during the easterly winds and fogs, it sunk even below 40°.

Fever, cynanche tonsillaris, catarrh, rheumatism, and hydrocephalus, seem to have been the prevailing diseases of the month.

The fevers, which were of the typhoid form, have been much more common than usual. They have appeared in almost every degree of severity, and although they seem to have principally affected infants and children, they have also been very common among those of maturer years.

The cases of cynanche tonsillaris, which have occurred in practice, have in some instances been very severe, and in others very slight. They probably owed their origin to the damp and foggy weather which prevailed during a great part of the month. Confinement to the house for a short time, with the use of gargles, cathartic medicines, and, in severe cases, with the application of a blister over the throat, effected a cure. When catarrh, which was by no means uncommon, existed along with cynanche, nearly the same sort of remedies removed both.

Rheumatism, both chronic and acute, has prevailed rather extensively. Acute rheumatism was commonly and speedily removed by the repeated use of sudorific medicines, and afterward by the use of friction to the affected part. Chronic rheumatism, like all other chronic complaints, compared with diseases in their acute state, has been more difficult of removal. The administration of mercury in various forms has given relief; arsenic also has produced some abatement of the symptoms. Indeed, these internally, and the frequent external application of friction with saponaceous balsam, mixed with tinctura opii, have been almost the only means of cure to which we have lately resorted.

I have lately met with various cases of hydrocephalus internus. It is much to be regretted, that the complaints of infants, and children in particular, have been rendered unnecessarily complex, not so much from their nature as from the preposterous catalogue of names which have been applied to them. Instead, however, of their being, as we have all been taught to believe, so very complex, they possess much more of one general character than the complaints of adults. This is a fortunate circumstance, as in the age of infancy, an expression of the feelings, indicative of certain states of disease, is not to be obtained.

It is owing to the mistaken views which I have alluded to, arising from our indolence of disposition, in taking for granted what has been said by others, rather than taking the trouble to think for ourselves, that multitudes of different, inefficacious, and often hurtful drugs, have been ridiculously administered in these cases, while the only simple, rational, and effectual plans of practice have been either neglected or too sparigly used to produce any beneficial effect.

This complaint, arising, almost in every instance, from a previous inflammatory state of the whole system, as may be ascertained by the pulse, the reduction of such a state is the grand object of the physician. For this purpose, it is almost always found sufficient, while we at the same time attend to the thoroughly clearing of the alimentary canal of dark coloured fæces, to make our applications as near the head as possible. Thus, leeches to the temples and a cap blister over the whole head, will, when attended to during the first two or three days, effect a cure in almost every instance.

P. S. I shall send some remarks, with cases, dissections, &c. for insertion in the Journal, in a short time.

*Princes Street.*

